# Analysis of the immunological biomarker profile during acute Zika virus infection reveals the overexpression of CXCL10, a chemokine linked to neuronal damage

**DOI:** 10.1590/0074-02760170542

**Published:** 2018-05-14

**Authors:** Felipe Gomes Naveca, Gemilson Soares Pontes, Aileen Yu-hen Chang, George Allan Villarouco da Silva, Valdinete Alves do Nascimento, Dana Cristina da Silva Monteiro, Marineide Souza da Silva, Lígia Fernandes Abdalla, João Hugo Abdalla Santos, Tatiana Amaral Pires de Almeida, Matilde del Carmen Contreras Mejía, Tirza Gabrielle Ramos de Mesquita, Helia Valeria de Souza Encarnação, Matheus de Souza Gomes, Laurence Rodrigues Amaral, Ana Carolina Campi-Azevedo, Jordana Graziela Coelho-dos-Reis, Lis Ribeiro do Vale Antonelli, Andréa Teixeira-Carvalho, Olindo Assis Martins-Filho, Rajendranath Ramasawmy

**Affiliations:** 1 Fundação Oswaldo Cruz-Fiocruz, Instituto Leônidas e Maria Deane, Programa de Pós-Graduação em Biologia da Interação Patógeno-Hospedeiro, Manaus, AM, Brasil; 2 Universidade Federal do Amazonas, Programa de Pós-Graduação em Imunologia Básica e Aplicada, Manaus, AM, Brasil; 3 Instituto Nacional de Pesquisas da Amazônia, Manaus, AM, Brasil; 4 George Washington University, Washington DC, United States of America; 5 Universidade do Estado do Amazonas, Manaus, AM, Brasil; 6 Hospital Adventista de Manaus, Manaus, AM, Brasil; 7 Fundação de Medicina Tropical Dr Heitor Vieira Dourado, Manaus, AM, Brasil; 8 Universidade Federal de Uberlândia, Patos de Minas, MG, Brasil; 9 Fundação Oswaldo Cruz-Fiocruz, Centro de Pesquisas René Rachou, Belo Horizonte, MG, Brasil; 10 Universidade Nilton Lins, Manaus, AM, Brasil

**Keywords:** Zika virus, CXCL10, biomarkers, chemokines, cytokines

## Abstract

**BACKGROUND:**

Infection with Zika virus (ZIKV) manifests in a broad spectrum of disease ranging from mild illness to severe neurological complications and little is known about Zika immunopathogenesis.

**OBJECTIVES:**

To define the immunologic biomarkers that correlate with acute ZIKV infection.

**METHODS:**

We characterized the levels of circulating cytokines, chemokines, and growth factors in 54 infected patients of both genders at five different time points after symptom onset using microbeads multiplex immunoassay; comparison to 100 age-matched controls was performed for statistical analysis and data mining.

**FINDINGS:**

ZIKV-infected patients present a striking systemic inflammatory response with high levels of pro-inflammatory mediators. Despite the strong inflammatory pattern, IL-1Ra and IL-4 are also induced during the acute infection. Interestingly, the inflammatory cytokines IL-1β, IL-13, IL-17, TNF-α, and IFN-γ; chemokines CXCL8, CCL2, CCL5; and the growth factor G-CSF, displayed a bimodal distribution accompanying viremia. While this is the first manuscript to document bimodal distributions of viremia in ZIKV infection, this has been documented in other viral infections, with a primary viremia peak during mild systemic disease and a secondary peak associated with distribution of the virus to organs and tissues.

**MAIN CONCLUSIONS:**

Biomarker network analysis demonstrated distinct dynamics in concurrence with the bimodal viremia profiles at different time points during ZIKV infection. Such a robust cytokine and chemokine response has been associated with blood-brain barrier permeability and neuroinvasiveness in other flaviviral infections. High-dimensional data analysis further identified CXCL10, a chemokine involved in foetal neuron apoptosis and Guillain-Barré syndrome, as the most promising biomarker of acute ZIKV infection for potential clinical application.

The Zika virus (ZIKV) is an arthropod-borne *Flavivirus*, transmitted mainly by female *Aedes* mosquitos and it that usually causes a mild illness characterized by conjunctivitis, pruritus, muscle and joint pain, rash, and slight fever. Outbreaks of ZIKV infection were first recorded in Micronesia and later in French Polynesia, where atypical manifestations were initially documented, including Guillain-Barré syndrome (Oehler et al. 2014). In Brazil, ZIKV infection during pregnancy has been linked to an unusual increase in the number of microcephaly cases (de Oliveira et al. 2016). Following the Brazilian report of congenital malformations, the number of microcephaly cases in French Polynesia was reanalysed, and a connection with ZIKV was further established. The broad spectrum of foetal clinical manifestations resulting from ZIKV infection led to a new classification termed congenital Zika syndrome.

The host immune response plays an important role in the clinical course of patients with viral infection. Particularly, cellular immunity and key components of the innate immune response, such as interferons and other cytokines/chemokines, play an essential role in limiting viral spread. To date, only two studies describing immune mediators in Zika-infected patients have been reported (Tappe et al. 2016, Kam et al. 2017). In Tappe et al. (2016), a reliable immunological biomarker profile during acute infection could not be established due to the small sample size. Kam et al. (2017) describes immune markers from a cohort from Campinas, Brazil, showing an inflammatory immune response and several immune mediators specifically higher in ZIKV-infected patients, with a statistically significant difference was observed in the levels of CXCL10, IL-10, and HGF observed between patients with and without neurological complications. Kam et al. (2017) also found higher levels of CXCL10, IL-22, MCP-1, and TNF-α in ZIKV-infected pregnant women carrying babies with foetal growth associated malformations.

In this study, we evaluated the immune response during acute ZIKV infection by analysing the serum levels of cytokines, chemokines, and growth factors from an adult cohort of 54 ZIKV-infected cases and 100 controls from Manaus, Brazil over five time points during symptomatic ZIKV infection. We present the time course of cytokine response in relation to viremia and identify a chemokine that may serve as a biomarker of acute ZIKV infection, thus providing new insights into ZIKV neuropathogenesis.

## MATERIALS AND METHODS

*Study population and design* - We used non-probabilistic convenience sampling and a cross-sectional experimental design, together with robust statistical analysis and data mining, for the evaluation of the immunological bio-marker profile during acute ZIKV infection. In the first half of 2016, a total of 54 suspected ZIKV-infected cases (29 non-pregnant females and 25 males, all adults) were recruited at Hospital Adventista de Manaus, Amazonas state, Brazil. All patients presented a maculopapular rash, with or without fever, and at least one of the following symptoms: pruritus, arthralgia, joint swelling, or conjunctival hyperemia within five days after symptom onset. Age-matched non-infected (NI) controls, 46 females and 54 males, were enrolled for comparison and basic characteristics, including data from physical examination and virological findings, were obtained. Comprehensive laboratory records, including routine laboratory tests, were available for 21 patients (15 male and six female).

*Ethics statement* - The study protocol was approved by the Ethics Committee of the Universidade do Estado do Amazonas (CAAE: 56745116.6.0000.5016), and all subjects provided written informed consent.

*Differential molecular diagnosis of Zika and viral load estimative* - Serum samples were sent to Fiocruz Amazônia and tested for ZIKV (envelope coding region) (Lanciotti et al. 2008), chikungunya virus (CHIKV) (Lanciotti et al. 2007), dengue virus (DENV) (Gurukumar et al. 2009), Mayaro virus (MAYV) and Oropouche virus (OROV) (Naveca et al. 2017) by real-time quantitative polymerase chain reaction (RT-qPCR). Samples positive for CHIKV, DENV, MAYV, or OROV were excluded from further analysis. Sample inclusion criteria also required the internal control (spiked MS2 bacteriophage) to display a Ct value between 30-32. The viremia was estimated by RT-qPCR using absolute quantification by the standard curve method and reported as viral RNA copies /mL.

*Dengue virus serology* - Serum samples were tested for previous exposure to DENV using Serion ELISA classic Dengue Virus IgG (Institut Virion/Serion GmbH, Germany).

*Microbeads assay for serum biomarkers* - A high-performance microbeads 27-plex assay (Bio-Rad, Hercules, CA, USA) was employed for detection and quantification of multiple targets, including: CXCL8 (IL-8), CXCL10 (IP-10), CCL11 (Eotaxin), CCL3 (MIP-1α), CCL4 (MIP-1β), CCL2 (MCP-1), CCL5 (RANTES), IL-1β, IL-6, TNF-α, IL-12, IFN-γ, IL-17, IL-1Ra (IL-1 receptor antagonist), IL-2, IL-4, IL-5, IL-7, IL-9, IL-10, IL-13, IL-15, FGF-basic, PDGF, VEGF, G-CSF, and GM-CSF. Samples were tested on a Bio-Plex 200 instrument (Bio-Rad) according to the manufacturer's instructions. The serum levels of IL-2, IL-7, and IL-15 were below the detection limits in several samples and were excluded from further analysis. The results were expressed as pg/mL.

*Statistical analysis and data mining* - Statistical analyses were initially performed using GraphPad Prism (GraphPad Software 6.0, San Diego, CA, USA). Comparative analysis of the clinical records was carried out using Fisher's exact test. The analysis of biomarker levels in NI controls vs*.* ZIKV-infected cases and between genders was performed using Mann-Whitney U tests. Multivariate correlations for biomarker levels and routine laboratory tests were analysed with the nonparametric Spearman's test (alpha .05) running on the JMP Software, v13.1.0 (SAS Institute, Cary, NC, USA). Correlations (Spearman r) were represented by a colour map matrix.

The dynamics of viremia, chemokines, cytokines, and growth factors were evaluated using the median value of each analyte. Comparative analysis of the bio-markers was carried out by Kruskal-Wallis test followed by Dunn's test. For all tests, differences were considered significant when p <.05 using two-tailed tests.

Data management strategies were applied to identify general and time-specific profiles. Biomarker signature analysis was carried out as previously described (Luiza-Silva et al. 2011). Radar charts were used to compile the biomarker signatures of NI controls and ZIKV-infected cases, applying the 75th percentile as the threshold. Venn diagrams were created to identify shared and unique attributes, along with the timeline of the symptoms onset (http://bioinformatics.psb.ugent.be/webtools/Venn/). Cytoscape software v3.2.0 (http://www.cytoscape.org/) was employed for visualizing and integrating multiple attributes into circular nodal networks. Connecting edges were drawn to underscore each association as positive (solid line) or negative (dashed line). The biomarker cluster pattern was defined by heatmaps assembled using R software (heatmap.2 function; v3.0.1). Decision tree algorithms were generated with WEKA software v3.6.11 (University of Waikato, New Zealand) to identify root and branch attributes and segregate patients from controls. ROC curves were built to define the cutoff and identify biomarkers with better performance in discriminating ZIKV-infected patients from NI controls. Performance indices (co-positivity, co-negativity, positive and negative likelihood ratios) were calculated using MedCalc software v7.3 (Ostend, Belgium).

## RESULTS

*Demographics, clinical records and virological data* - The 54 Brazilian Zika cases, 29 non-pregnant females (median age 38 years, interquartile range (IQR) 27.5 −46.5) and 25 males (median age 37 years, IQR 30 - 50), were enrolled between the first and fifth day after symptom onset. A group of 100 non-infected control subjects who were residents of Manaus, Amazonas, Brazil were also included (46 females (median age 28 years, IQR 23 - 36) and 54 males (median age 29.5 years, IQR 23 −36). The median viremia level, expressed as copies/mL, was 2,031 (minimum = 133, maximum = 2.4 x 10^6^, IQR: 881 - 5,268). The frequency of specific ZIKV symptoms was similar between men and women, with the exception that men had an increased frequency of fever compared to women (100% versus 67%, p = 0.005) (Table I). DENV IgG testing showed that 94.4% (51/54) of the patients were positive, two had an undetermined result, and one male subject was negative.

**TABLE I:** Demographical aspects, clinical records and virological status of Zika virus (ZIKV)-infected patients

Parameters		All	Females	Males	p
Non-infected controls					
	n	100	46	54	NA
	Age (years)	29.0 (23-36)	28.0 (23-36)	29. 5 (23-36)	0.58
ZIKV-infected patients					
	n	54	29	25	n.a.
	Age (years)	37. 5 (29 - 4 8)	38.0 (27.5-46.5)	37.0 (30 −5 0)	0.79
	Days of symptoms onset	2.5 (2-4)	2.0 (1- 4)	3.0 (2-4)	0.32
	Rash	95.0%	94.4%	95.5%	1.0 0
	Fever	85.0%	66.7%	100.0%	**0.005**
	Myalgia	82.5%	83.3%	81.8%	1.0 0
	Conjunctival hyperemia	75.0%	66.7%	81.8%	0.30
	Pruritus	70.0%	66.7%	72.7%	0.73
	Headache	65.0%	66.7%	63.6%	1.00
	Arthralgia	60.0%	72.2%	50.0%	0.20
	Joint swelling	25.0%	33.3%	18.2%	0.30
	Vomiting or nausea	25.0%	33.3%	18.2%	0.30
	Diarrhea	17. 5%	27. 8%	9.1%	0. 21
	Lymphadenopathy	12.5%	11.1%	13.6%	1.0 0
	Viremia (ZIKV RNA copies/mL)	2,0 31 (8 81-5, 26 8)	2,130 (1,078-5,268)	1,786 (802-8,720)	0.77

Data are reported as median and interquartile range (IQR) for age, days of symptoms onset and viremia. Statistical differences were assessed by Mann-Whitney test. Comparative analysis of clinical records observed in females and males was carried out by Fisher's exact test. Significant differences were considered at p < 0.05 for comparisons between females *vs*. males and are underscored by **bold/underlined** format. Viremia is expressed as copies /mL as described in material and methods. NA: not applicable.

**Fig. 1:**
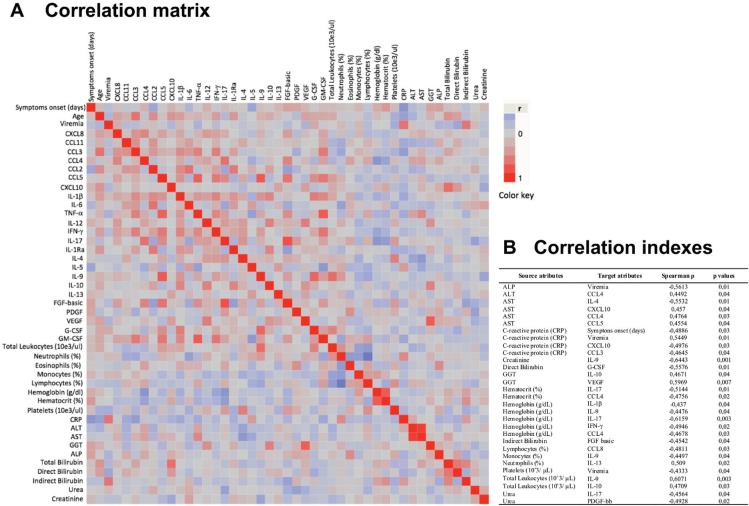
immunological biomarker correlations with the results of routine laboratory tests, age, viremia, and symptoms. The nonparametric Spearman's test was applied to evaluate multiple correlations between immunological biomarkers and the results of routine laboratory tests. A colour map matrix was plotted showing the strength and direction of these correlations (-1 blue, to +1 red), panel A. Statistically significant correlations (p < .05) between immunological biomarkers and routine tests are highlighted in the inserted table, panel B.

*Correlation of immunological biomarkers with routine laboratory tests during acute ZIKV infection* - The data on 45 continuous variables including immunological biomarkers, routine laboratory tests, age, viremia, and symptoms onset were analysed (Fig. 1A). Overall, moderate correlations were observed for several variables, and the strongest correlations were observed between TNF-a and CCL5 (Spearman r = 0.8245), and lymphocytes (%) and neutrophils (%) (Spearman r = 0.8084). All results were represented in a colour map matrix, where statistically supported associations (p <.05) between routine laboratorial tests and immunological biomarkers were highlighted (Fig. 1B).

**Fig. 2:**
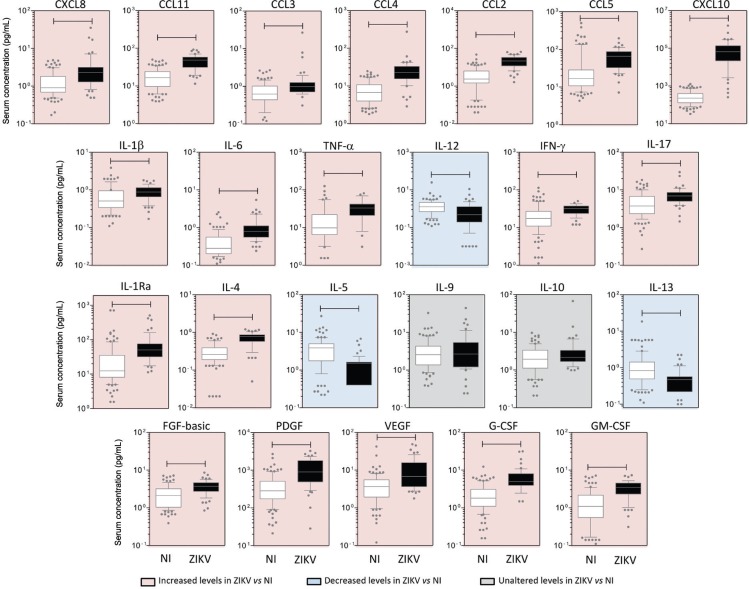
panoramic overview of serum chemokines, cytokines, and growth factors during early stages of Zika virus (ZIKV) infection in adults. Serum biomarkers (CXCL8, CCL11, CCL3, CCL4, CCL2, CCL5, CXCL10, IL-1β, IL-6, TNF-α, IL-12, IFN-γ, IL-17, IL-1Ra, IL-4, IL-5, IL-9, IL-10, IL-13, FGF-basic, PDGF, VEGF, G-CSF, and GM-CSF) were measured in ZIKV-infected patients (D1 to D5, ZIKV = 
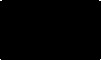
, n = 54) and non-infected subjects [non-infected (NI) = 
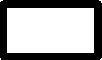
, n = 100] by high performance Luminex 27-plex assay as described in Methods. Data are expressed as pg/mL and are displayed in box and whisker (10-90 percentile) plots. Comparative analysis between NI vs. ZIKV was performed by Mann-Whitney test and significant differences at p < .05 are underscored by connecting lines. Coloured backgrounds highlight increased (pink), decreased (blue), and unaltered (grey) levels of serum biomarkers in ZIKV as compared to NI.

*ZIKV-infected patients display high levels of circulating biomarkers* - Elevated levels of pro-inflammatory cytokines (IL-1β, IL-6, TNF-α, IFN-g and IL-17, except IL-12, which was higher in controls), chemokines (CXCL8, CCL11, CCL3, CCL4, CCL2, CCL5, and CXCL10) and growth factors (FGF-basic, PDGF, VEGF, G-CSF, and GM-CSF) were found in ZIKV-infected cases (Fig. 2, pink panels), whereas higher levels of IL-5 and IL-13 were seen in controls (Fig. 2, blue panels). Interestingly, the levels of IL-4 and IL-1Ra were also higher among patients as compared to controls. No differences were observed for IL-9 and IL-10 (Fig. 2, grey panels). A similar pattern was observed when results were stratified by gender, although infected males presented significant lower levels of CCL3, CCL4, CCL5, IL-17, FGF-basic, and GM-CSF than females. No significant differences were observed between female and male controls (Table II).

**TABLE II:** Serum chemokines, cytokines and growth factors early after Zika virus (ZIKV) infection (Dl to D5) in adult females and males

	Females (F) n = 29			Males (M) n = 25				Score ZIKV/NI	
Analytes	NI	ZIKV	P(l)	NI	ZIKV	P(2)	P(3)	(F)	(M)
CXCL8	0.87(0.54-1.67)	2.26 (1.57-3.26)	**0.0001**	0.98 (0.70-1.93)	2.30(1.19-3.11)	**0.0018**	0.5669	2.6	2.3
CCL11	16.44 (9.29-22.22)	48.94(30.12-61.91)	**0.0001**	16.65 (10.62-25.20)	43.82 (29.11-56.95)	**0.0001**	0.3488	3.0	2.6
CCL3	0.59 (0.41-0.86)	1.15 (0.80-1.32)	**0.0001**	0.65 (0.45-1.13)	0.89 (0.67-1.07)	**0.0469**	**0.0205**	1.9	1.4
CCL4	7.28 (4.56-12.20)	28.76 (18.76-35.67)	**0.0001**	5.94 (3.75-9.79)	20.18 (12.63-26.64)	**0.0001**	**0.0162**	4.0	3.4
CCL2	2.08(1.00-4.97)	20.73 (13.45-34.70)	**0.0001**	2.46 (1.94-715)	21.98(11.86-32.34)	**0.0001**	0.9862	10.0	8.9
CCL5	15.17(11.36-34.98)	82.77 (64.75-108.00)	**0.0001**	17.00 (9.83-25.70)	34.06 (23.66-65.81)	**0.0001**	**0.0001**	5.5	2.0
CXCL10	232 (128-434)	71,219 (32,899-148,407)	**0.0001**	218 (109-392)	44,645 (10,423-69,757)	**0.0001**	0.1030	307	205
IL-1β	0.52 (0.24-0.96)	0.93 (0.56-1.19)	**0.0176**	0.52 (0.29-1.00)	0.77(0.61-1.14)	0.1511	0.5374	1.8	1.5
IL-6	0.29 (0.20-0.57)	0.79 (0.63-1.00)	**0.0001**	0.28 (0.21-0.55)	0.81 (0.52-1.73)	**0.0001**	0.1944	2.7	2.9
TNF-α	9.76 (6.10-20.20)	35.87 (25.45-44.08)	**0.0001**	10.08 (6.45-22.62)	26.93(15.02-41.84)	**0.0015**	0.1101	3.7	2.7
IL-12	1.26 (0.51-2.30)	0.63 (0.22-1.66)	0.0554	1.39 (0.96-2.17)	0.34 (0.09-1.08)	0.0001	0.1895	0.5	0.2
IFN-γ	14.97 (9.63-24.54)	31.91 (26.39-38.65)	**0.0001**	19.79 (14.14-2731)	26.41 (23.61-35.97)	**0.0035**	0.4283	2.1	1.3
IL-17	3.84 (2.21-753)	7.88 (6.28-9.02)	**0.0001**	3.57 (2.50-6.76)	5.96 (4.41-715)	**0.0114**	**0.0092**	2.1	1.7
IL-1Ra	11.81 (781-28.66)	47.00 (34.03-65.59)	**0.0001**	12.33 (8.95-36.13)	54.94 (30.77-115.10)	**0.0001**	0.3623	4.0	4.5
IL-4	0.27(0.20-0.39)	0.81 (0.59-0.86)	**0.0001**	0.26 (0.17-0.41)	0.73 (0.53-0.90)	**0.0001**	0.6890	3.0	2.8
IL-5	3.16 (1.67-5.02)	1.50(0.40-1.61)	**0.0001**	4.70 (2.00-5.35)	1.38(1.07-1.61)	**0.0001**	0.5792	0.5	0.3
IL-9	2.21 (1.19-4.11)	3.10 (1.69-5.72)	0.0783	2.62 (1.59-4.69)	1.42 (1.18-3.83)	0.0837	0.0518	1.4	0.5
IL-10	1.73 (0.75-3.39)	2.11 (1.65-3.22)	0.1046	1.95 (1.51-3.02)	2.25 (1.52-3.41)	0.5478	0.9571	1.2	1.2
IL-13	0.75(0.37-1.34)	0.48 (0.22-0.57)	**0.0135**	0.98 (0.80-1.57)	0.57(0.37-0.57)	**0.0001**	0.3634	0.6	0.6
FGF-basic	1.84(1.01-3.14)	4.34 (3.71-5.27)	**0.0001**	2.24 (1.28-3.73)	3.24 (2.13-4.24)	**0.0468**	**0.0014**	2.4	1.4
PDGF	359 (125-585)	1,012 (616-1,933)	**0.0001**	258 (196-403)	823 (416-1,578)	**0.0001**	0.3670	2.8	3.2
VEGF	2.87(1.78-6.29)	6.72 (4.18-16.33)	**0.0001**	3.70 (2.17-4.97)	6.26 (3.66-15.21)	**0.0005**	0.5668	2.3	1.7
G-CSF	1.86 (1.04-2.86)	5.96 (4.43-8.05)	**0.0001**	1.83 (1.19-3.29)	4.94 (3.42-766)	**0.0001**	0.2400	3.2	2.7
GM-CSF	0.87(0.52-2.00)	3.76 (3.03-4.64)	**0.0001**	1.35 (0.54-2.16)	2.88(1.73-3.91)	**0.0003**	**0.0256**	4.4	2.1

Data are reported as median levels (IQR) in pg/mL. Statistical analysis was performed by Mann-Whitney test and significance reported as p-values: p(l), p(2), and p(3) for comparisons between non-infected (NI) *vs*. ZIKV females, NI *vs*. ZIKV males and ZIKV females *vs*. ZIKV males, respectively. Significant differences between NI *vs*. ZIKV are underscored in **bold** format. Differences between ZIKV females *vs*. ZIKV males are highlighted by **bold-underlined** format. No significant differences were observed between NI females *vs*. NI males. Score represents the fold change (analyte median value in infected patient divided by analyte median value in controls) segregated by gender.

**Fig. 3:**
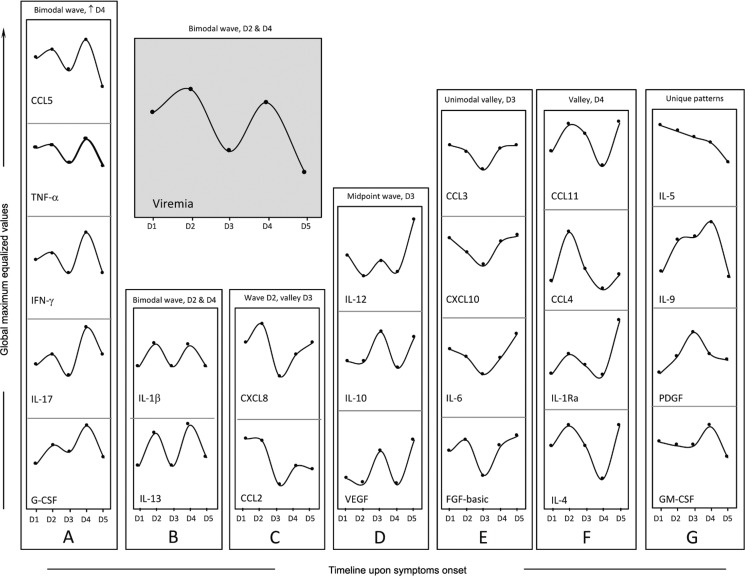
rhythms of viremia, chemokines, cytokines, and growth factors during early stages of Zika virus (ZIKV) infection in adults. Cross-sectional follow-up of viremia and serum biomarkers was carried out in ZIKV-infected patients categorized according to the time (days) of symptom onset (D1, n = 11; D2, n = 13; D3, n = 10; D4 n = 9 and D5 n = 5). Viremia displayed a bimodal profile with similar waves at D2 and D4 (grey panel). Distinct patterns were identified for clusters of biomarkers, as they displayed kinetic curves shaping a bimodal wave at D2 and a higher wave [↑] at D4 (panel A: CCL5, TNF-α, IFN-γ, IL-17, and G-CSF); a bimodal profile with similar waves at D2 and D4 (panel B: IL-1β and IL-13); a wave at D2 and a valley at D3 (panel C: CXCL8 and CCL2); a midpoint wave at D3 (panel D: IL-12, IL-10, and VEGF); a unimodal valley at D3 (panel E: CCL3, CXCL10, IL-6, and FGF-basic); a valley at D4 (panel F: CCL11, CCL4, IL-1Ra, and IL-4) or a unique pattern (panel G: IL-5, IL-9, PDGF, and GM-CSF). Data are displayed as global maximum equalized median values of the serum concentrations (pg/mL) for each biomarker.

*Bimodal viremia is accompanied by increased levels of a defined group of biomarkers* - Viremia and biomarker levels were assessed at different time points (day 1 upon symptoms onset was denoted as D1, etc.), with D1 (n = 11), D2 (n = 13), D3 (n = 10), D4 (n = 09), and D5 (n = 05). A bimodal distribution was observed, with two viremia peaks at D2 and D4, with the lowest viremia levels at D5 (Fig. 3, grey panel). Dynamics of CCL5, TNF-α, IFN-γ, IL-17, and G-CSF were closely related to viremia (Fig. 3A). A similar bimodal distribution was observed for IL-1b and IL-13 (Fig. 3B). The highest levels of CXCL8 and CCL2 were observed at D1 and D2 (Fig. 3C). An inverse correlation was observed for IL-12, IL-10, and VEGF (Fig. 3D), where the highest levels were observed at the lowest levels of viremia. The levels of CCL3, CXCL10, IL-6, and FGF-basic displayed a distinct pattern, with the lowest levels observed at D3, coinciding with the first drop in viremia (Fig. 3E). A valley at D4 followed by an increase at D5 was observed for CCL11, CCL4, IL-1Ra, and IL-4 (Fig. 3F), and unique patterns were observed for IL-5, IL-9, PDGF, and GM-CSF (Fig. 3G).

**Fig. 4:**
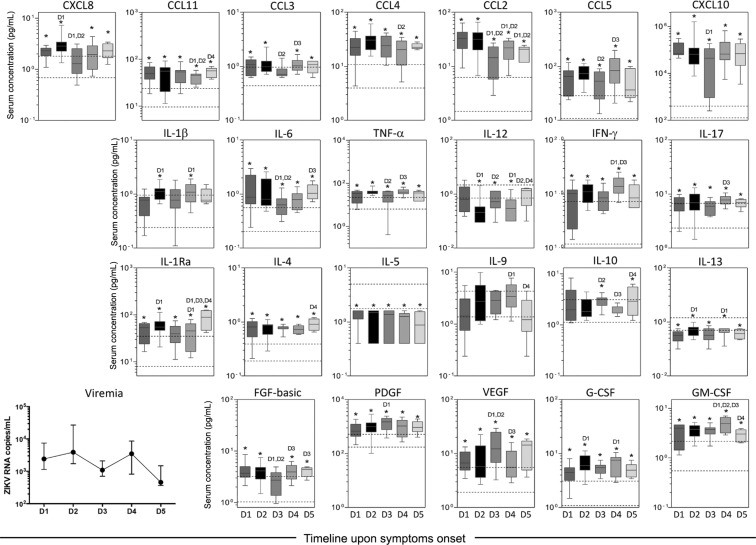
kinetics of viremia, serum chemokines, cytokines, and growth factors during early stages of Zika virus (ZIKV) infection in adults. Cross-sectional analysis of viremia and serum biomarkers was performed in ZIKV-infected patients categorized according to the time (days) of symptom onset (D1, n = 11; D2, n = 13; D3, n = 10; D4, n = 09 and D5, n = 05). ZIKV RNA copies/mL are displayed as the median and interquartile range (IQR). Biomarker data are expressed in pg/mL and are displayed in box and whisker (10-90 percentile) plots. Multiple comparisons amongst distinct time points of symptom onset were performed by Kruskal-Wallis test followed by Dunn's post-test. Significant differences at p < .05 were identified at D1, D2, D3, and D4 as compared to day 1, day 2, day 3, and day 4, respectively. Analysis was also carried out by Mann-Whitney test to compare each time point from ZIKV-infected patients with those from a single group of non-infected controls (NI). Significant differences at p < .05 are marked by asterisks (*). Reference ranges for each biomarker were established as interquartile ranges (25th-75th percentiles) observed in NI (dashed lines). Distinct patterns were identified for clusters of biomarkers, as they displayed kinetic curves shaping a bimodal wave at D2 and a higher wave [↑] at D4 (CCL-5, TNF-α, IFN-γ, IL-17, and G-CSF), a bimodal profile with similar waves at D2 and D4 (IL-1β and IL-13), a wave at D2 and a valley at D3 (CXCL8 and CCL2), a midpoint wave at D3 (IL-12, IL-10, and VEGF), a unimodal valley at D3 (CCL3, CXCL10, IL-6, and FGF-basic), a valley at D4 (CCL11, CCL4, IL-1Ra, and IL-4) or a unique pattern (IL-5, IL-9, PDGF, and GM-CSF).

Biomarkers were also evaluated in controls, and the IQR are represented by dashed lines (Fig. 4). Most bio-marker levels differed between patients and controls at all time points, except for IL-10 at D1 and D2, and IL-1b at D3. No differences were observed for IL-9.

**Fig. 5:**
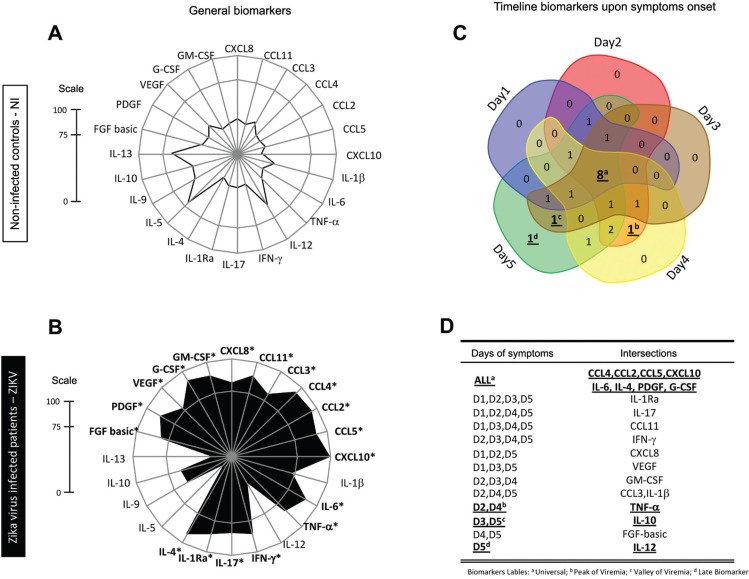
biomarker levels upon symptom onset and along a time course during early stage Zika virus (ZIKV) infection in adults. Biomarker signatures of non-infected (NI) (
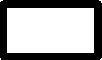
) and ZIKV (
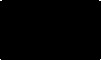
) were constructed as described in Methods. Data are presented in radar charts as the proportion of subjects with serum biomarker levels above the global population median values in (A) NI subjects, and (B) ZIKV-infected patients. Biomarkers with levels above the global median in more than 75% of subjects were highlighted by asterisks (*). (C) A Venn diagram showing the intersections of common attributes and well as selected biomarkers along the timeline of symptom onset: day 1 (blue), day 2 (red), day 3 (brown), day 4 (yellow), and day 5 (green). (D) Venn diagram report summarizing selected attributes with patterns labelled as (a) universal, (b) peak of viremia, (c) valley of viremia, or (d) late biomarkers (inserted table).

*ZIKV infection elicited a set of general and timeline-specific biomarkers* - The biomarker levels were used to build a signature (Fig. 5A-B) as described in the Methods section. A significant difference in the overall profile was observed in ZIKV-infected cases as compared to controls. Furthermore, the radar chart revealed that 19/24 (79%) biomarkers were highly induced by ZIKV infection. Almost all biomarkers analysed were found at levels above the global median in more than 75% of the infected patients (Fig. 5, B panel).

Venn diagram analysis showed that four chemokines (CCL4, CCL2, CCL5, CXCL10), two cytokines (IL-6, IL-4), and two growth factors (PDGF, G-CSF) were significantly induced at all time points (Fig. 5C). Of note, TNF-α appears as the only biomarker at the intersection of the viremia peaks (D2 and D4). In contrast, IL-10 is the only unregulated biomarker at viremia valleys (D3 and D5), while increased levels of IL-12 appear at D5 (Fig. 5D).

**Fig. 6:**
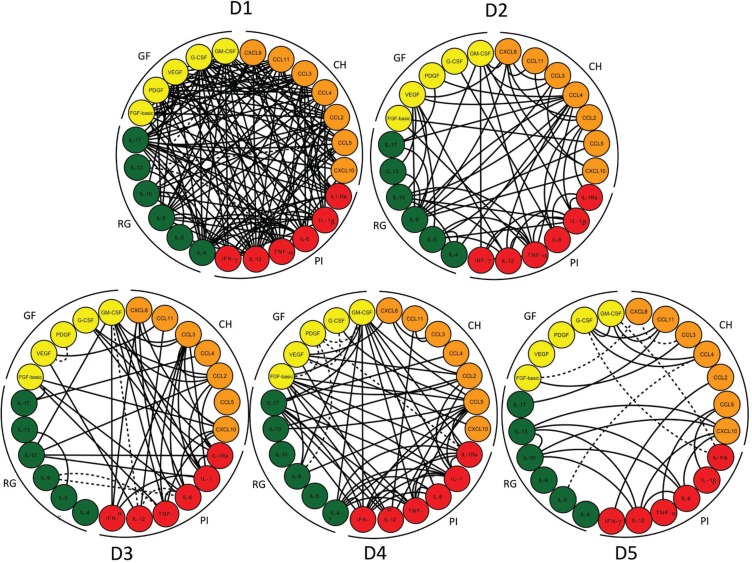
biomarker networks along a timeline during early stages of Zika virus (ZIKV) infection in adults. Integrative systems biology analysis of attributes was conducted using the Cytoscape software platform to build a circular nodal network layout for each time point following ZIKV infection, from day 1 (D1) up to day 5 (D5), based on Spearman's correlation matrices. Significance was considered at p < .05. The timeline of networks is displayed as circular layouts to characterize the interaction along the early time points. Coloured nodes are employed to identify chemokines (CH - orange), pro-inflammatory cytokines (PI - red), regulatory cytokines (RG - green), and growth factors (GF - yellow). Connecting edges underscore the association between attributes, classified as positive (solid line) or negative (dashed line).

*Distinct biomarker networks are observed at different time points* - Cytoscape software was used to conduct a correlative analysis of immunological biomarkers. The exploratory analysis demonstrated that earlier infection was associated with more complex biomarker networks. Most correlations at D1 and all correlations at D2 were positive (solid lines). The level of complexity decreased from D1 to D5 (Fig. 6).

**Fig. 7:**
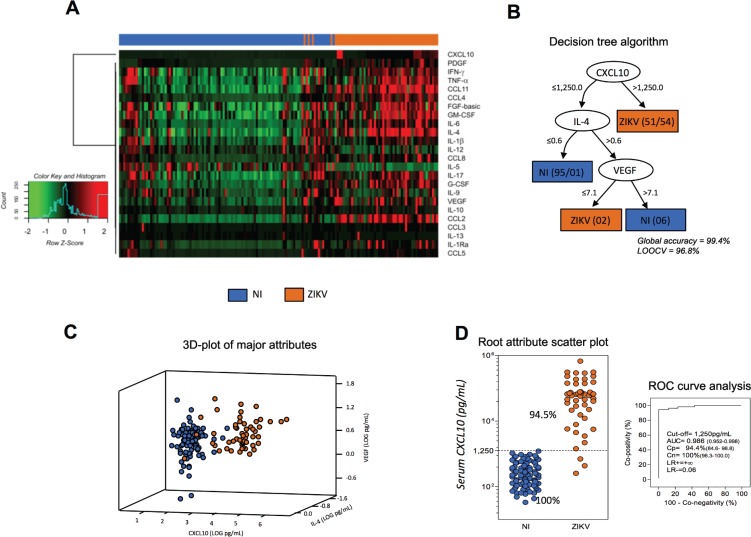
high-dimensional data analysis during early stages of Zika virus (ZIKV) infection in adults. Machine-learning high-dimensional data approaches were applied to further explore and identify feasible criteria applicable for the clinical follow-up of ZIKV infection. (A) Heatmap panels were built to verify the ability of attributes to segregate ZIKV (orange) and non-infected (NI) (blue) groups as they present low (green) or high (red) levels of serum biomarkers. (B) Decision tree algorithms were generated to define root and branch attributes to segregate patients (ZIKV = orange) from NI controls (NI = blue). Global accuracy and leave-one-out cross-validation (LOOCV) values are provided in the figure. (C) The root/branch attributes selected by the decision tree algorithm were compiled into a 3D-plot to verify their cluster strengths. (D) The performance of the selected root attribute to discriminate ZIKV (orange) from NI (blue) was evaluated by scatterplot distribution and validated by receiver operating characteristic indices (AUC: area under the curve; Cp: co-positivity; Cn: co-negativity; LR+/LR-: positive/negative likelihood ratio).

*High-dimensional data analysis identified CXCL10 as the most promising biomarker for a putative clinical application* - A heatmap matrix was constructed to evaluate the profile of biomarkers associated with ZIKV infection. CXCL10 clustered with one clade separately from the other attributes (Fig. 7A). In addition, a decision tree was built to identify the biomarker most able to segregate patients. This approach confirmed the heatmap observations indicating CXCL10 as the most specific biomarker, followed by IL-4 and VEGF. The analysis showed a very high global accuracy (99.4%) with a leave-one-out cross-validation of 96.8% (Fig. 7B). The significance of these attributes (CXCL10, IL-4, and VEGF) was assessed by 3D-plots, and the performance of the root attribute (CXCL10) was evaluated by scatterplot distribution and ROC curve analysis (Fig. 7C-D). CXCL10 alone showed a very high global accuracy ranging from 0.952-0.998. Together, the results demonstrated that CXCL10 measurement identified 94% of the patients, with no false positive identification and outstanding indices (co-positivity, co-negativity, and likelihood ratio).

## DISCUSSION

The pathogenesis of ZIKV infection is still largely unknown, and the main determinants of disease manifestations are not yet well established. Understanding serum immunomodulators during acute infection may be a first step in elucidating the mechanisms underlying ZIKV-induced immunopathology.

We show that the immune response during the acute phase of ZIKV infection is polyfunctional and broadly inflammatory, as evidenced by significantly elevated levels of IL-4, IL-17, IFN-γ, IL-1β, IL-1Ra, TNF-α, and IL-6 in patients as compared to controls. This is consistent with findings from Kam et al. (2017) that a robust pro-inflammatory cytokine response occurs during acute ZIKV infection, with elevations of IL-18, TNF-α, IFN-γ, IL-8, IL-6, GRO-α, and IL-7. A polyfunctional immune activation associated with increased CCL2, CXCL10, IL-6, IL-8, VEGF, and G-CSF levels but decreased levels of IL-13 was also described in the amniotic fluid of ZIKV-positive pregnant women whose infants had microcephaly (Ornelas et al. 2017). Additionally, other studies found higher serum levels of IL-5 and IL-13 amongst healthy controls as compared to infected individuals (Galliez et al. 2016, Kam et al. 2017). Both IL-5 and IL-13 are effector molecules essential to type 2-inflammation, especially in atopic asthma and viral respiratory tract infections (Edwards et al. 2017). However, the role of these cytokines in the ZIKV-host response is still unclear.

When we stratified the results by gender, ZIKV-infected males presented lower levels of CCL3, CCL4, CCL5, IL-17, FGF-basic, and GM-CSF. The reason for this difference is unknown in the context of ZIKV infection; however, this finding is consistent with the literature demonstrating that females tend to mount a higher innate and adaptive immune system response to viruses as compared to men (Klein 2012). In addition, this discrepancy could be due to females being sampled on average one day earlier than men, with a median time from onset to diagnostic sampling of two days for females and three days for males.

It is possible that previous exposure to flavivirus antigens may affect the immune response to ZIKV infection. In the present study, almost all patients (51/54) tested positive for DENV IgG antibodies. Manaus has had several dengue epidemics, including co-circulation of different serotypes (Figueiredo et al. 2008). Moreover, the Amazonas state is endemic for the yellow fever virus (YFV) and has very high YFV-vaccination coverage. Thus, most individuals enrolled in this study experienced previous flavivirus exposure potentially modulating the cytokine and chemokine responses. These differences in prior flavivirus exposure may account for some differences in the cytokine and chemokine profiles shown in the study by Kam et al. (2017), which examined a Brazilian cohort of patients from Campinas, Brazil, where yellow fever vaccination was not required by the government at the time of their study as it is in Manaus, Brazil.

Consistent with our results, an immune response induced during the acute phase has previously been described in infections caused by ZIKV and other flaviviruses, including YFV and West Nile virus (ter Meulen et al. 2004, Klein et al. 2005, Tappe et al. 2016). In the case of ZIKV infection, the mechanism of the inflammatory immune response has not been clearly delineated. The immune response may be triggered by viral upregulation of the expression of pattern recognition receptors (PRRs) engaged in downstream pathways and the inflammatory antiviral response, such as IRF7, IFN-α, IFN-β, and CCL5 (Hamel et al. 2015). Interestingly, we showed a strong positive correlation between IFN-α and CCL5, suggesting that the synergistic effect of these cytokines might be crucial for the outcome of acute inflammation caused by ZIKV.

Our findings also revealed higher levels of growth factors and chemokines among patients as compared to controls. Similarly, prior research showed increased levels of CXCL10, CCL5, CCL3, and VEGF in patients acutely infected with ZIKV, while elevated levels of GMCSF, CCL4, and FGF-basic biomarkers were observed only in the recovery phase (Tappe et al. 2016). Our study demonstrated that all chemokines and growth factors analysed were significantly increased in the acute phase when compared with non-infected controls. In fact, the role of growth factors in the pathogenesis of arboviral infections remains a matter of debate. We demonstrate that a remarkable increase of FGF-basic, PDGF, VEGF, G-CSF, and GM-CSF identifies the acute phase of ZIKV infection, which suggests the importance of chemokines and growth factors in the initiation and regulation of the acute phase immune response.

Increased serum concentrations of both CXCL (CXCL8 and CXCL10) and CCL chemokines (CCL2, CCL3, CCL4, CCL5, and CCL11) were found in acute ZIKV infection. The role of CCL5 in arbovirus-induced immunopathology remains a controversial issue, but levels of this chemokine, along with CCL2 and CCL3, were previously linked to the severity of dengue virus infections, including neurological disease and impairment of neuronal survival (Sathupan et al. 2007, Zlotnik and Yoshie 2012).

Furthermore, we found strong correlations between TNF-a and CCL5 concentrations and the percentages of circulating neutrophils and lymphocytes in acute ZIKV infection. This finding is likely due to the role of TNF-a and CCL5 in leukocyte chemoattraction and demonstrates the important role of this cytokine and chemokine in the stimulation of the innate and adaptive immune system in response to ZIKV infection.

This manuscript is the first to describe the bimodal nature of viremia in acute Zika infection and the corresponding peaks in inflammatory cytokine production. A biological model explaining bimodal viremia was first described in a classical study of using mousepox virus (Fenner 1948). Similarly, flaviviruses are initially replicated in Langerhans cells at the site of inoculation and in draining regional lymph nodes. Despite a robust antiviral innate immune response that eliminates viral infected cells, some virus particles are disseminated by the blood (primary viremia). Therefore, several organs and tissues may become infected, producing a second wave of viral replication that reaches the blood and causes secondary viremia. The equine infection by African horse sickness virus, another arbovirus of the *Orbivirus* genus, *Reoviridae* family, also shows two viremia peaks. The first peak is observed after viral multiplication in lymph nodes, whereas the second peak is observed after viral replication in spleen, lungs and endothelial cells (Mellor and Hamblin 2004).

Interestingly, bimodal viremia has been found in patients after low dose live attenuated 17DD yellow fever vaccine administration (Campi-Azevedo et al. 2014). As compared to the standard dosage vaccine, the low dose live attenuated vaccine is hypothesized to elicit a less robust immune response that does not clear the initial viremia, leading to a second peak of viremia a few days later. Although bimodal viremia, including in flavivirus infections, was observed in the aforementioned studies, our results should be interpreted with caution, as we did not evaluate patients longitudinally. Thus, future studies on this topic are recommended.

In this manuscript, we report high levels of pro-inflammatory mediators during the acute phase of ZIKV infection. Paradoxically, although the inflammatory response leads to viral clearance, the high levels of circulating pro-inflammatory biomarkers may facilitate the transmission of viruses from the circulation to the central nervous system by increasing the permeability of the blood-brain barrier. This phenomenon has been reported for the West Nile virus (Wang et al. 2004), as well as another neurovirulent flavivirus, and may partially explain ZIKV neuroinvasiveness.

Remarkably, CXCL10 expression was increased more than 200-fold in ZIKV-infected subjects as compared to controls. This CXCL10 overexpression has been linked to the IFN-γ signalling pathway induced by ZIKV NS5 protein. According to Chaudhary et al. (2017), NS5 promotes IFN-γ gene activation through the degradation of STAT2 and subsequent induction of STAT1-STAT1 homodimerization. Augmented serum levels of CXCL10 have been found during severe clinical manifestations of dengue and yellow fever (Melchjorsen et al. 2003). Surprisingly, CXCL10 has also been shown to play an important role in CD-8+ T-cell recruitment as part of an anti-flaviviral response to West Nile virus in the central nervous system (Klein et al. 2005). Furthermore, CXCL10 has been previously identified as a biomarker of severity in several diseases including those caused by bacteria such as *Mycobacterium tuberculosis* and *Legionella pneumophila*, as well as protozoans like *Trypanosoma brucei*, *Leishmania major*, *Plasmodium vivax* or *Plasmodium falciparum* (Liu et al. 2011). Other studies showed that the overexpression of CXCL10 leads to apoptosis in foetal neurons (Liu et al. 2011). CXCL10 has also been strongly implicated in Guillain-Barré syndrome pathogenesis (Chiang and Ubogu 2013). Thus, we hypothesize that the high levels of CXCL10 in ZIKV patients may contribute to neuronal damage affecting the developing foetal brain and potentially targeting peripheral nerves in Guillain-Barré syndrome as well. Consistent with this hypothesis, Kam et al. (2017) specifically identified higher levels of CXCL10 in ZIKV-infected patients with neurological complications compared to those without and higher levels of CXCL10 in ZIKV-infected pregnant women carrying babies with foetal growth associated malformations.

High levels of CXCL10 have been previously described in acute and convalescent phases, with more prominent expression in the latter (Tappe et al. 2016). Unfortunately, although our data strongly suggest that CXCL10 is a biomarker of acute ZIKV infection, we were unable to perform a longitudinal analysis to verify its kinetics in order to further confirm whether the concentrations of this chemokine would be down or up-regulated across different stages of the disease. In addition, CXCL10 elevation is also observed in pre-eclampsia and hypertension in pregnancy, which can result in a range of foetal injuries including intrauterine growth retardation and neurological damage induced by hypoxia (Gotsch et al. 2007). Thus, it is reasonable to suggest that ZIKV-induced inflammation may increase the frequency of foetal injuries.

CXCL10 may also be an important therapeutic target (Liu et al. 2011). For example, CXCL10 neutralization by specific antibodies or genetic deletion in CXCL10-/-mice protected against cerebral malaria infection and inflammation (Nie et al. 2009). Passive transfer of anti-CXCL10 antibodies reduced inflammatory leukocyte recruitment across the blood-brain barrier. Furthermore, statin medications commonly used for cholesterol control have been shown to decrease CXCL10 and to be effective in CXCL10-mediated Crohn's disease (Grip and Janciauskiene 2009).

In this work, we also describe the relationship between the timing of viremia and cytokine elevations. We assessed the acute phase biomarkers and viral titres at different time points (until day 5). Augmented levels of CCL4, CCL2, CCL5, CXCL5, CXCL10, IL-6, IL-4, PDGF, and G-CSF immunomodulators were observed at all time points. The peaks of viremia, at Day 2 and Day 4, were accompanied by increased TNF-α levels. IL-10 elevation appeared to be directly related to the lowest virus titres (Day 3 and Day 5), while the highest levels of IL-12 were found at Day 5. These findings allow us to deduce that the acute phase of ZIKV is characterized mainly by an innate immune system inflammatory response, with overlap of the inflammatory biomarkers and viremia peaks, while the anti-inflammatory response coincides with viremia decay. Altogether, this study identifies unique characteristics of the acute inflammatory and multifactorial immune response induced by ZIKV and identifies CXCL10 as a potential biomarker of acute infection and, perhaps, a predictor of severity. Nevertheless, further longitudinal studies that measure the host immunopathological response at several time points are required to better characterize the immunological factors involved in Zika disease. The altered concentrations of serum biomarkers observed in this study may bring new insights to the ZIKV immunopathology puzzle.
